# Characterization of Multiscale Regulation of the Brain in Aging and Age-related Conditions: A Comprehensive Review

**DOI:** 10.1093/geroni/igaf122.2270

**Published:** 2025-12-31

**Authors:** Haoru Li, Brad Manor, Lewis Lipsitz, Junhong Zhou

**Affiliations:** Vanderbilt University, Nashville, Tennessee, United States; Hebrew SeniorLife/Harvard Medical School, Boston, Massachusetts, United States; Hebrew Senior Life, Boston, Massachusetts, United States; Harvard Medical School Hebrew SeniorLife, Roslindale, Massachusetts, United States

## Abstract

As the core for many important functions, the brain activities are regulated by numerous underlying neuro-biophysiological elements communicating and interacting over multiple temporospatial scales. Studies have emerged to characterize such complex multiscale regulation of the brain (i.e., brain complexity), especially under the influences of aging and age-related conditions/diseases, via advanced signal-processing techniques, and obtained exciting insights into the pathology of brain-related neurodegenerative diseases that cannot be characterized using traditional single-scale measures. We thus performed a literature review to comprehensively summarize these achievements/progress in this area. Seven electronic databases were searched. The key information (e.g., participant cohort) of 43 eligible studies was extracted. These studies were all cross-sectionally designed with relatively small sample size. The brain signals in older adults with (n = 40) (i.e., Parkinson’s disease (n = 5), Alzheimer’s disease (n = 31) and other types of conditions (n = 4)) and without (n = 3) age-related conditions were recorded using electroencephalogram (n = 23), functional MRI (n = 10) and near-infrared-spectroscopy (n = 2), and magnetoencephalography (n = 8). The brain complexity was measured by multiscale entropy, transfer entropy (TE) or partial TE (i.e., quantifying the efficiency of the information exchange between brain regions). Compared to younger-age control or those with normal aging, individuals in older age and/or with age-related conditions had altered multiscale regulation of the brain (e.g., lower brain complexity), and lower complexity was associated with advanced disease stage and poorer functional performance. The evidence level was relatively low. It is thus highly demanded to confirm these findings in studies with larger sample size, and explore the causal relationship between brain complexity and functions.

